# Supplementary material for: Resolving the full spectrum of human genome variation using Linked-Reads
Source: Genome Res. 2019 Apr;29(4):635–45. doi: 10.1101/gr.234443.118 (PMC6442396; doi:10.1101/gr.234443.118)
Supplement: Supplemental Material [file supp_gr.234443.118_Supplemental_longranger-2.2.zip › longranger-2.2.tar.bz2!/lr2.2-public/lib/stan/cmdstan/stan/doc/api/html/common_8hpp_source.html]

Stan: src/stan/common.hpp Source File


|  |  |
| --- | --- |
|  | Stan  2.5.0  probability, sampling & optimization |


- Main Page
- Related Pages
- Namespaces
- Classes
- Files

- File List
- File Members

All Classes Namespaces Files Functions Variables Typedefs Enumerations Enumerator Friends Macros Pages

- src
- stan

common.hpp

Go to the documentation of this file.

1 #ifndef STAN\_\_COMMON\_HPP\_

2 #define STAN\_\_COMMON\_HPP\_

3

4 #include <stan/common/command.hpp>

5 #include <stan/common/do\_print.hpp>

6 #include <stan/common/init\_adapt.hpp>

7 #include <stan/common/init\_nuts.hpp>

8 #include <stan/common/init\_static\_hmc.hpp>

9 #include <stan/common/init\_windowed\_adapt.hpp>

10 #include <stan/common/print\_progress.hpp>

11 #include <stan/common/run\_markov\_chain.hpp>

12 #include <stan/common/recorder.hpp>

13 #include <stan/common/sample.hpp>

14 #include <stan/common/warmup.hpp>

15 #include <stan/common/write\_error\_msg.hpp>

16 #include <stan/common/write\_iteration.hpp>

17 #include <stan/common/write\_iteration\_csv.hpp>

18 #include <stan/common/write\_model.hpp>

19 #include <stan/common/write\_stan.hpp>

20

21 #endif

write\_iteration\_csv.hpp

warmup.hpp

run\_markov\_chain.hpp

write\_stan.hpp

init\_nuts.hpp

write\_model.hpp

print\_progress.hpp

write\_error\_msg.hpp

recorder.hpp

sample.hpp

write\_iteration.hpp

command.hpp

do\_print.hpp

init\_windowed\_adapt.hpp

init\_adapt.hpp

init\_static\_hmc.hpp


---

[ Stan Home Page ]

*© 2011–2014,
Stan Development Team.*
